# Controlling exchange bias in Fe_3_O_4_/FeO composite particles prepared by pulsed laser irradiation

**DOI:** 10.1186/1556-276X-6-226

**Published:** 2011-03-16

**Authors:** Zaneta Swiatkowska-Warkocka, Kenji Kawaguchi, Hongqiang Wang, Yukiko Katou, Naoto Koshizaki

**Affiliations:** 1 Nanosystem Research Institute, National Institute of Advanced Industrial Science and Technology (AIST), 1-1-1 Higashi, Tsukuba, 305-8565 Ibaraki, Japan

## Abstract

Spherical iron oxide nanocomposite particles composed of magnetite and wustite have been successfully synthesized using a novel method of pulsed laser irradiation in ethyl acetate. Both the size and the composition of nanocomposite particles are controlled by laser irradiation condition. Through tuning the laser fluence, the Fe_3_O_4_/FeO phase ratio can be precisely controlled, and the magnetic properties of final products can also be regulated. This work presents a successful example of the fabrication of ferro (ferri) (FM)/antiferromagnetic (AFM) systems with high chemical stability. The results show this novel simple method as widely extendable to various FM/AFM nanocomposite systems.

## Introduction

Magnetic nanoparticles and hybrid magnetic nanostructures are of growing interest because of their technological applications in magnetic recording media, sensitive magnetic sensors and various biomedical applications such as drug delivery system, hyperthermia or magnetic resonance imaging [1]. In order to fulfil the requirements of many of these applications, an accurate control over the coercivity is strongly required.

Exchange bias coupling at the ferro (ferri) (FM)/antiferromagnetic (AFM) interface has attracted considerable attention due to their applications in permanent magnet applications and high density recording media [2,3]. The exchange bias effect is manifested by the shifting and broadening of a magnetic hysteresis loop of a sample cooled under an applied field [1,4,5]. Although the intrinsic origin of exchange bias effect is not yet understood fully, it is generally accepted that the interface exchange coupling between FM and AFM is the origin of the exchange bias [6].

Exchange bias has been extensively studied in bilayer and multilayer thin films [7,8], nanoparticles with core/shell structure [9-11] and particles dispersed in matrix [12]. However, to date, the report about how to control the exchange bias by changing the FM/AFM ratio is seldom.

So far, various experimental methods have been used to produce FM/AFM heterostructure particles, e.g. chemical and thermal decomposition [10,13,14], ball milling method [11], gas condensation and chemical vapour deposition [15,16]. However, decomposition methods need the chemicals which often cannot be removed and remain as residual molecules on particle surfaces. Gas-phase methods require expensive and large-scale vacuum equipments. Such methods are generally effective for preparing particles with a narrow size distribution. However, most of these approaches are limited to synthesizing particles with a diameter smaller than 30 nm. Additionally, most of mentioned methods lead to the sintering of two phases and the poor quality of the interface. This has been attributed to the weak interfacial interaction between the FM and AFM phases in particles and results in a weak exchange bias. Therefore, the development of new synthetic techniques for FM/AFM particles with high exchange bias is still a target of current research.

In this study, we demonstrate a novel method for preparing submicrometer iron oxide nanocomposite spherical particles by pulsed laser irradiation in liquid (PLIL). In contrast to the pulsed laser ablation in liquid using a focused laser beam, which has been widely studied, PLIL irradiating source particles dispersed in liquid with an unfocused laser light which gives relatively mild reaction conditions [17,18]. The present study demonstrates the easy control of size and composition of submicrometer spherical iron oxide particles by PLIL. Furthermore, we report the structural effect and the exchange bias effect in Fe_3_O_4_/FeO composites by PLIL method. Varying the phase ratio of magnetite and wustite, we can control the coercivity and exchange bias effect.

## Experimental

The magnetite nanoparticles were prepared by conventional co-precipitation from FeCl_2 _and FeCl_3 _at high values of pH. Iron salts were dissolved in water with a magnetic stirrer for 1 h. The pH value was increased by adding NaOH. The colour of the solution turned to black immediately, inducting magnetite formation. Magnetite particles were removed from the solution by using permanent magnet and were washed several times with deionized water. Finally, the magnetite nanoparticles were dispersed in ethyl acetate and transferred to a sealed cell with quartz window to introduce laser light. The magnetite nanoparticles were stirred and irradiated for 1 h with the third harmonic (355 nm) of an Nd:YAG (yttrium aluminium garnet) laser operated at 30 Hz without focusing. Laser fluence varied from 33 to 177 mJ/pulse cm^2^. No evaporation of solvents was observed during irradiation.

The formed iron oxide phases and composition were determined by a powder X-ray diffractometer (XRD) (Rigaku Ultima IV, Rigaku Corporation, Akishima, Tokyo, Japan) with CuKα radiation. The morphology of the obtained particles was observed by a field emission scanning electron microscope (FE-SEM) (Hitachi S4800, Hitachi High Technologies Japan Inc., Tokyo, Japan) and a transmission electron microscope (TEM) (JEOL JEM 2010, Tokyo, Japan). Average particle size was determined by measuring the diameters of 200 particles from SEM images. The size of spherical particles was simply defined from the diameter. Calculation of size of non-spherical particles was based on replacing a given particle with a sphere that has the same volume as a given particle. The chemical states of elements in the samples were confirmed by an X-ray photoelectron spectrometer (XPS) (PHI, Versa Probe, ULVAC-PHI, Inc., Chigasaki, Kanagawa, Japan). A highly sensitive superconducting quantum interference device (Quantum Design, MPMS, San Diego, CA, USA) magnetometer was employed to measure the magnetic properties of nanocomposite particles. Hysteresis measurements were recorded for dried samples of nanoparticles in a gelatin capsule. Hysteresis loops were obtained by using maximum applied field up to 50 kOe at 5 and 300 K. The exchange bias properties of samples were investigated by measuring field cooled (FC) hysteresis loops in the temperature range 5-300 K. In the FC procedure, the sample was cooled down from the initial temperature of 300 K to the measuring temperature *T*, under an applied field 50 kOe. Once *T *was reached, the field was set to 50 kOe and the measurement of the loop started.

## Results and discussion

### Fabrication and structural investigation of Fe_3_O_4_/FeO system obtained by PLIL method

**Figure 1:**
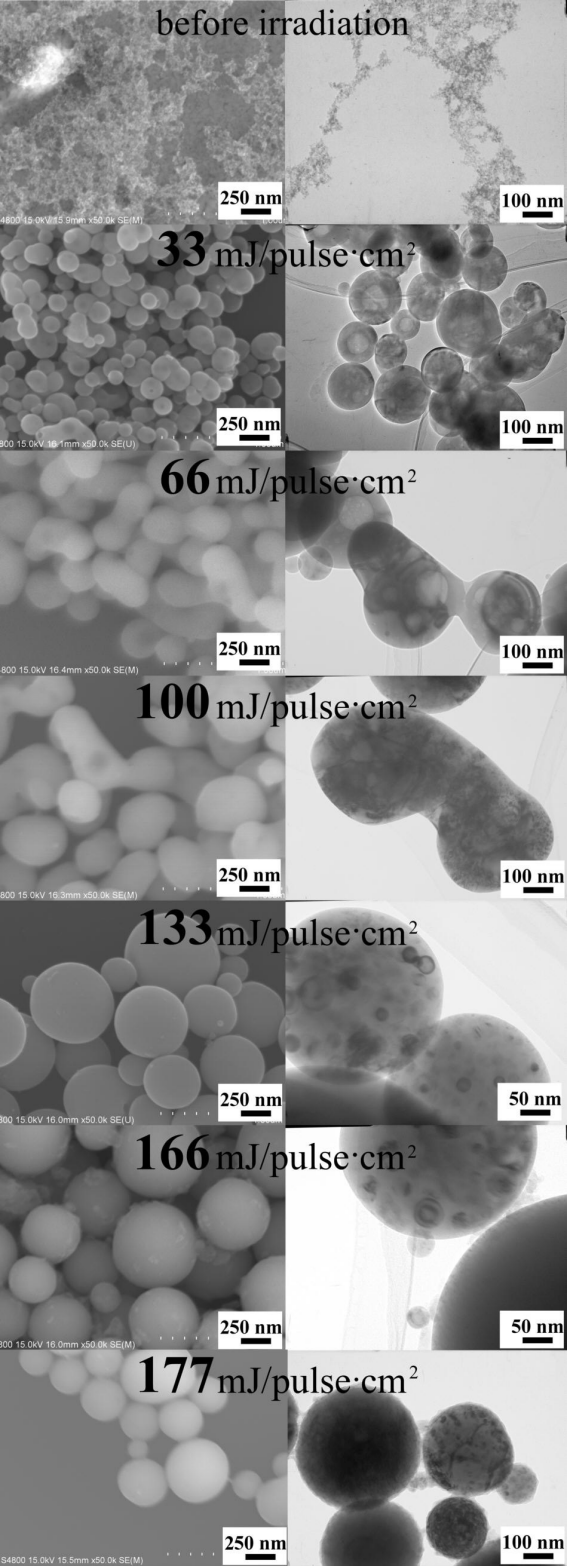
**FE-SEM and TEM images of iron oxide nanoparticles**. Before and after laser irradiation with various fluences.

**Figure 2:**
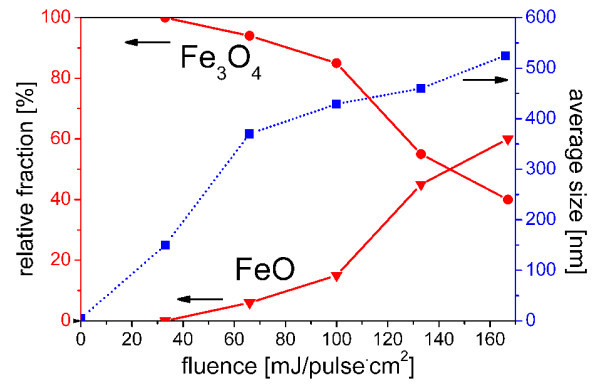
**Examination by FE-SEM**. Variation of particle size (*dotted curve*) and relative fraction of Fe_3_O_4 _and FeO with fluence (*solid line*).

The size and shape of the particles obtained by laser irradiation in ethyl acetate were examined by FE-SEM (Figure 1, left). The average diameter of raw magnetite nanoparticles in the aggregates (Figure 1 before irradiation) is estimated to be 6 nm. Figure 1 indicates that spherical particles with smooth surfaces were formed after laser irradiation. Their spherical shape clearly indicates melt formation during the process, which suggests that the temperature of the particles is transiently increased over the melting point of iron oxide. A fluence increases from 33 to 177 mJ/pulse cm^2 ^and shows a systematic increase in the particle size from 150 to 460 nm (Figures 1 and 2).

The relationship between particle size and fluence is simply explained by the thermal energy absorbed of laser light. The absorption cross section of particles with diameters larger than the irradiation laser wavelength is considered the same as the geometrical cross section. The minimum energy to melt a particle is proportional to the particle volume (∝ *d*^3^), while the absorption energy is proportional to the particle's cross section (∝ *d*^2^). Thus, the minimum fluence to melt a particle is proportional to the diameter *d *= *d*^3^/*d*^2^. The relationship, however, is not so simple for particles with a diameter equal to or less than laser wavelength because of the complex dependence of the cross section on particle size [18,19].

TEM analyses provided more detailed structural information on the submicrometer spheres (Figure 1, right). Some of the particles formed at 33-66 mJ/pulse cm^2 ^had hollow structures. In contrast, smaller particles ranging from 5 to 60 nm were embedded in the larger spherical particles at a fluence exceeding 100 mJ/pulse cm^2^. In the intermediate fluence range of 66-100 mJ/pulse cm^2^, particles with a merged structure of two primary particles were observed.

**Figure 3:**
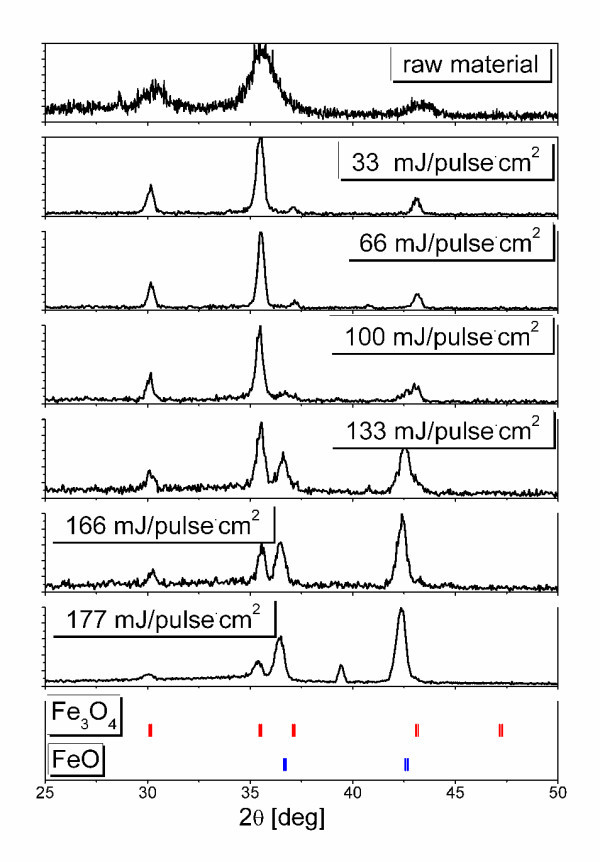
**X-ray diffraction patterns of raw magnetite and irradiated nanoparticles at various laser fluencies**. Standard XRD peaks for Fe_3_O_4 _and FeO are plotted for reference.

X-ray diffraction patterns of particles before and after laser irradiation (Figure 3) revealed a gradual phase transformation from magnetite (Fe_3_O_4_) to wustite (FeO) with fluence increase. Starting raw nanoparticles were confirmed to be a pure magnetite phase. The sample after irradiation at 33 mJ/pulse cm^2 ^remained pure magnetite without chemical change, though the crystalline size increased judging from the reduced width and increased intensity of the reflections. Small wustite reflections of (111) at 36.1 and (200) at 42.0 appears as shoulders of magnetite (222) at 37.1 and (400) at 43.1, respectively in the 66-mJ/pulse cm^2 ^result in Figure 3. Those wustite reflections grew and magnetite ones decreased with the irradiation fluence increase. Volume fractions of Fe_3_O_4_/FeO, calculated by ratio of highest intensity peaks from XRD data, are summarized in Figure 2.

**Figure 4:**
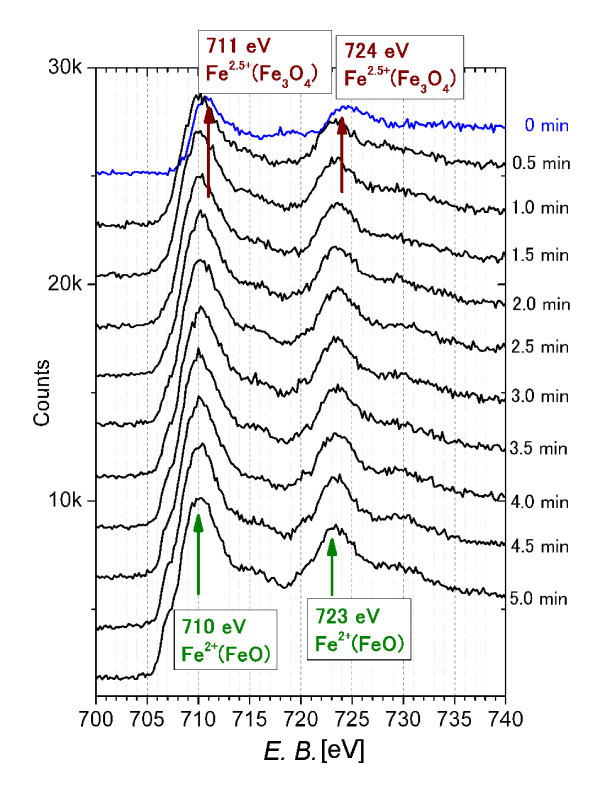
**XPS Fe 2p depth profile of the Fe_3_O_4_/FeO particles fabricated at 177 mJ/pulse cm^2^**.

Further information about the iron oxide formation during laser irradiation can be gained by analyzing XPS data. For the sake of simplicity, just the XPS Fe 2p depth profile of sample obtained after irradiation with fluence 177 mJ/pulse cm^2 ^is shown as being representative of the behaviour of all composite particles (Figure 4). All spectra for pre-sputtering and post-sputtering shows peaks positioned around 711 and 724 eV, which are typical core level spectra of Fe_3_O_4 _and around 710 and 723 eV, which are characteristic for Fe^2+ ^ions in FeO [20]. These results are qualitatively consistent to the XRD measurements. On the basis of TEM and XPS results, we can suppose that composite particles obtained by pulsed laser irradiation reveal aggregated structures.

Morphology and composition of the particles obtained by laser irradiation suggest that Fe_3_O_4 _nanoparticles are melted to form a large spherical shape and reduced to form FeO phase. Temperature to melt iron oxide nanoparticles definitely induces the decomposition of surrounding ethyl acetate and possibly leads to the reduction of magnetite to wustite. Thermodynamic calculation was performed to investigate the possible thermal decomposition reaction of ethyl acetate and probable reducing reaction of magnetite. Gibbs free energy calculation of possible thermal decomposition reaction suggests that ethyl acetate can be thermodynamically decomposed at 1,600°C (the melting point of bulk magnetite) to methane, ethylene, carbon monoxide or hydrogen, and that these gases can reduce Fe_3_O_4 _to FeO.

The magnetite nanoparticles dispersed in ethyl acetate melt and formed spherical hollow particles by laser irradiation at low fluence. The formation of submicrometer hollow particles at low fluence may be related to the confining process of bubbles by melted droplets. Such bubbles may result from ultrasonic stirring during irradiation. With laser fluence increase, the reducing reaction with the decomposed gases becomes significant. Partial surface melting of particles causes coalescence with close neighbours (in the intermediate fluence range) and/or formation of spherical composite particles (in the high fluence range), together with the reducing reaction by decomposed gas from ethyl acetate. Thus, a hundred nanometer-sized particles composed of magnetite and wustite nanoparticles grow with increased fluence.

### Magnetic properties of Fe_3_O_4_/FeO system with different Fe_3_O_4 _to FeO phase ratios

In order to investigate the impact of different phase ratio of magnetite and wustite on the magnetic properties of the final Fe_3_O_4_/FeO composite, the Fe_3_O_4 _nanoparticles dispersed in ethyl acetate were irradiated with the fluence of 133, 166 and 177 mJ/pulse cm^2 ^for 0.5, 1 and 2 h. The obtained particles are spherical with FeO volume fraction that varies from 20% to 85%. All particles have similar structures to those presented in Figure 1 with the fluence of 133 mJ/pulse cm^2 ^or larger.

**Figure 5:**
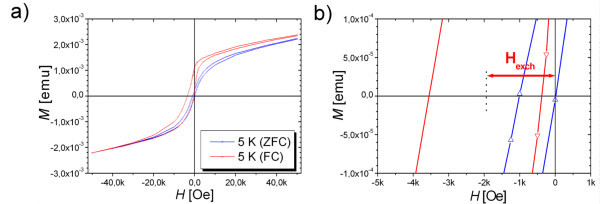
**Illustration of the FC and ZFC hysteresis loops**. **(a)** Hysteresis loops of the Fe_3_O_4_/FeO particles fabricated at 177 mJ/pulse^.^cm^2^. FC means that the sample is cooled from 300 to 5 K in the 50 kOe field. **(b) **The magnification around origin of hysteresis loops of the Fe_3_O_4_/FeO particles fabricated at 177 mJ/pulse cm^2^.

Exchange coupling at the FM/AFM interface of the Fe_3_O_4_/FeO system is investigated by the zero field cooled (ZFC) and field cooled (FC) measurements of *M*(*H*). Figure 5 illustrates the FC (*H*_FC _= 50 kOe) and ZFC hysteresis loops at 5 K for a cycling field of ± 50 kOe of sample with 75% of wustite fraction (the ZFC and field cooled (FC) measurements of *M*(*H*) curves for particles with 20%, 45%, 60% and 85% of FeO are presented in supporting information on Figure S1 in Additional file 1). The interesting feature in the *M*(*H*) curves is that both the ZFC and FC loops remain open even in the 50 kOe field, known as the high field irreversibility, which could be interpreted as being due to the existence of the spin glass-like (SGL) phase [21,22]. According to the figure, this system exhibits the properties of exchange bias system, with a horizontal shift along the field axis of the FC hysteresis loop with respect to the ZFC hysteresis loop. The loop shift is defined as an exchange bias field *H*_exch _= |(*H*^+ ^+ *H*^-^)/2|, where *H*^+ ^and *H*^- ^are positive and negative coercive fields. The FC hysteresis loop is shifted with an exchange bias field of 1,960 Oe. The coercivity field given by *H*_c _= (*H*^+ ^- *H*^-^)/2 is also obtained for both the ZFC case with the value of 514 Oe and a considerably higher value of 1950 Oe for FC cases. The large coercivity and exchange bias indicates a strong magnetic interaction through the interface between magnetite and wustite. Additionally, a slight positive vertical shift along the magnetization axis is presented. In FM/AFM systems, vertical shifts are generally related to pinned uncompensated spins that exhibit either FM or AFM coupling at the interface [23]. The positive vertical shift in Figure 5 indicates a dominant FM coupling between the pinned uncompensated spins and the FM magnetization.

**Figure 6:**
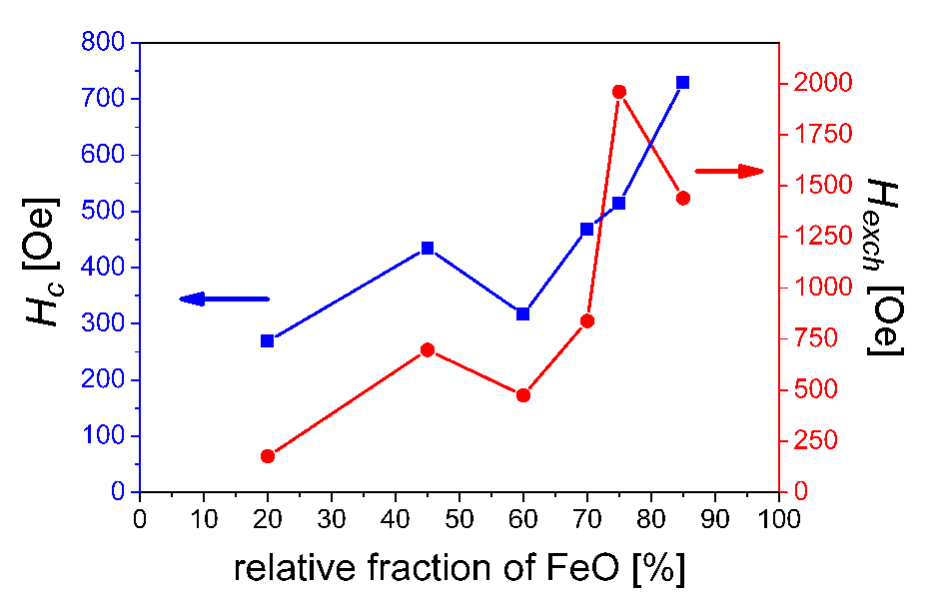
**Variation of *H*_c _and *H*_exch_**. Coercivity *H*_c _and exchange bias *H*_exch _of Fe_3_O_4_/FeO composite particles as a function of relative fraction of FeO in particles measured at 5 K.

To reveal the effect of the phase ratio of FM and AFM phase on the exchange bias of FM/AFM composites, *H*_c _and *H*_exch _as a function of FeO phase ratio in Fe_3_O_4_/FeO particles was investigated. Figure 6 shows the variation of *H*_c _(after ZFC) and *H*_exch _(after FC in 50 kOe) with the increase FeO fraction in particles at 5 K. It is clear that *H*_c _increases with the increasing FeO fraction, while *H*_exch _firstly increases and then reaches a maximum of 1,960 Oe for 75% of wustite fraction. Further increase of FeO concentration leads to the decrease of *H*_exch_. With increasing FeO concentration, the AFM phase can supply enough force to pin the uncompensated spin in FM phase which leads to a much stronger exchange interaction and further increase in *H*_exch_. With a further increase in FeO concentration (> 75%), the balance between FM and AFM is destroyed, and *H*_exch _do not increase continuously with increasing FeO concentration. In this case, 75% is a critical concentration of AF phase in Fe_3_O_4_/FeO composites. This result can confirm granular structure of obtained particles. With increasing FeO phase the effective interface area increases affecting exchange bias. AFM is too high for 75% of FeO concentration, and the effective interface area rapidly decreases entailing the exchange bias decrease

**Figure 7:**
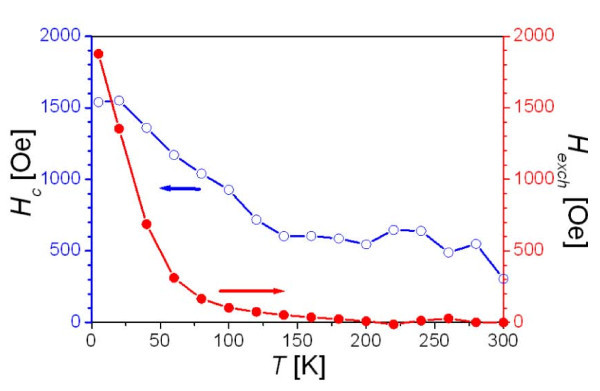
**Magnetic hysteresis loops for the samples with 75% of FeO**. Coercivity *H*_c _and exchange bias *H*_exch _of Fe_3_O_4_/FeO composite particles as a function of temperature.

In order to explore the origin of *H*_exch_, the temperature dependence of *H*_exch _obtained from magnetic hysteresis loops for the samples with 75% of FeO were also studied. The sample was first cooled down from 300 K to the measuring temperature under a magnetic field of 50 kOe, and then the loop was measured. This process was repeated for every measuring temperature. As presented in Figure 7, *H*_exch _decreases with the temperature increase and appears to vanish at about 190 K. This blocking temperature is equal to the Neel temperature (*T_N_*) of FeO (for FeO *T_N _*= 198 K [24]). At a higher temperature, the coupling between FM and AFM regions is weakened by thermal disturbance. As the temperature decreases, the exchange interaction between the above two types of regions becomes stronger, resulting in the loop shift which becomes more prominent at a lower temperature. However, coercivity *H*_c _does not decrease to zero and its value approaches to the intrinsic *H*_c _of particles without exchange bias.

Thus, the observed exchange bias effect can be explored on the exchange coupling between the interfacial FM phase and AFM (or SGL) phase, and AFM can play an important role in pinning the uncompensated interfacial moments.

## Conclusions

In conclusion, the pulsed laser irradiation technique was demonstrated to be a simple method for preparing submicrometer iron oxide heterostructure spherical particles. Size and composition of obtained particles can be tuned in a controllable manner by only laser fluence. Additionally, obtained particles exhibit interesting magnetic properties, especially exchange bias interaction at the ferrimagnet-antiferromagnet interface. For 75% of AFM concentration, the *H*_exch _can reach the maximum value 1,960 Oe, at 5 K after field cooling. The reason is that the FM and AFM phase reach to the balance; the value of pinning force of AFM phase, which can play a significant role in pinning the uncompensated interfacial moments, is maximum. *H*_exch _decreases with increasing temperature and approach zero at 190 K. The exchange bias originates from the exchange coupling between the interfacial FM phase and AFM phase. Although generally core/shell structures have been considered to explain a large exchange bias field, we have developed a new type of nanocomposite system with a large exchange bias field composed of ferrimagnetic Fe_3_O_4 _and antiferromagnetic FeO by pulsed laser irradiation of colloidal nanoparticles.

In contrast with common chemical methods, pulsed laser irradiation in liquid is very simple, low-cost, and contamination-free. Hence, we believe that our method makes it possible to synthesize magnetic heterostructure particles with controllable size, composition and magnetic properties.

## Competing interests

The authors declare that they have no competing interests.

## Authors' contributions

ZS-W conducted most of the experiments and performed data analysis. KK supported the magnetic property measurement and contributed the data interpretation. HW supported to construct the formation mechanism by providing relating data of similar systems. YK helped the most of operation and data interpretation of analytical equipments used. NK conceived basic idea of this technique and supported the organization of this paper.

## Supplementary Material

Additional file 1 **supporting information**. Fig. S1 Magnetization vs. field loop measured at 5 K under ZFC and FC conditions Fe_3_O_4_/FeO composite particles with different fraction of FeO. Click here for file
