# Supplementary material for: Controlling exchange bias in Fe3O4/FeO composite particles prepared by pulsed laser irradiation
Source: Nanoscale Res Lett. 2011 Mar 16;6(1):226. doi: 10.1186/1556-276X-6-226 (PMC3211284; doi:10.1186/1556-276X-6-226)
Supplement: Additional file 1 — supporting information. Fig. S1 Magnetization vs. field loop measured at 5 K under ZFC and FC conditions Fe3O4/FeO composite particles with different fraction of FeO. [file 1556-276X-6-226-S1.PDF]

Supporting information

Controlling exchange bias in Fe_3O_4/FeO composite particles prepared by pulsed laser irradiation

Zaneta Swiatkowska-Warkocka[1§], Kenji Kawaguchi[1], Hongqiang Wang[1], Yukiko Katou[1] and Naoto Koshizaki[1]

[1] Nanosystem Research Institute, National Institute of Advanced Industrial Science and Technology (AIST), 1-1-1 Higashi, Tsukuba, 305-8565 Ibaraki, Japan

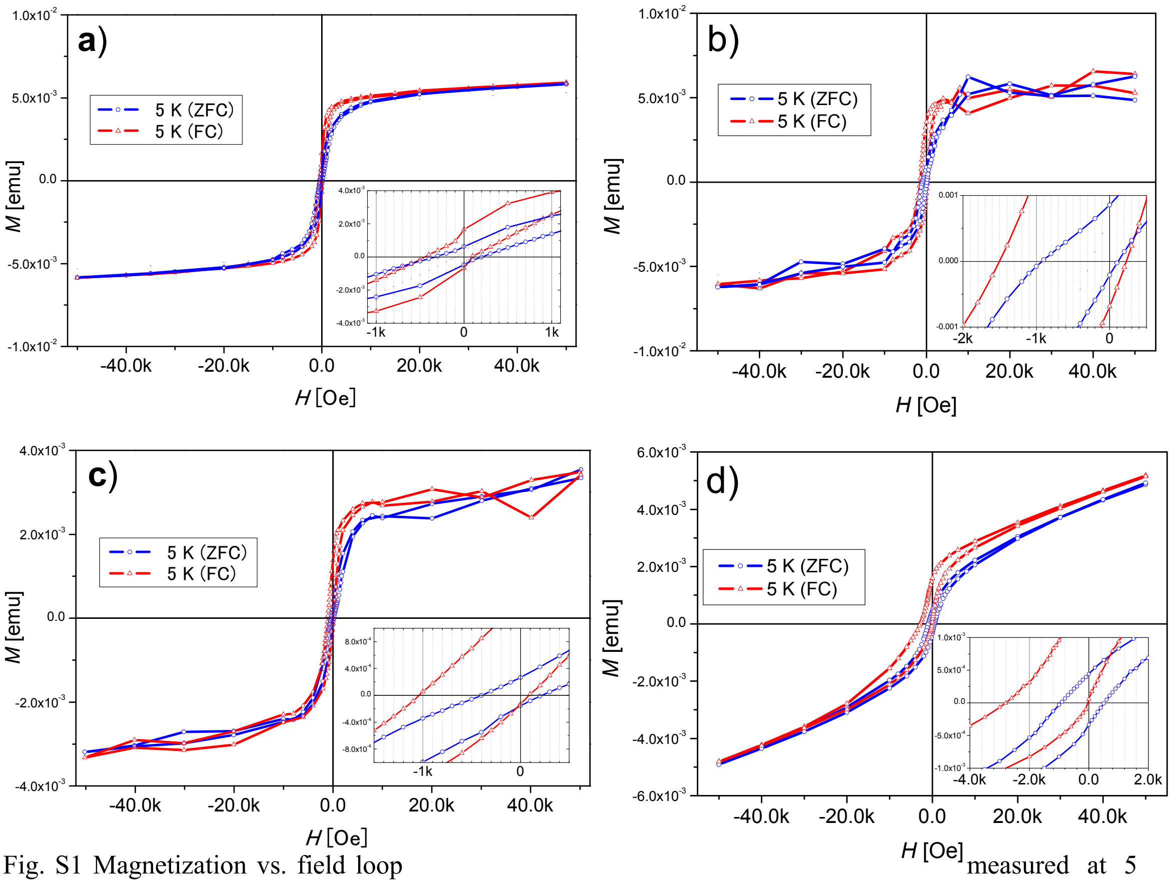

Fig. S1 Magnetization vs. field loop measured at 5 K under ZFC and FC conditions Fe_3O_4/FeO composite particles with different fraction of FeO: (a) 20%, (b) 45%, (c) 60% and (d) 85%. The insets show an enlarged view of the low field region.
